# The Changing Face of Esophageal Cancer 

**DOI:** 10.3390/cancers2031379

**Published:** 2010-06-28

**Authors:** Rachel E. Melhado, Derek Alderson, Olga Tucker

**Affiliations:** Academic Department of Surgery, Queen Elizabeth Hospital, University Hospitals Birmingham, Birmingham, UK; E-Mails: Derek.Alderson@uhb.nhs.uk (D.A.); Olga.Tucker@uhb.nhs.uk (O.T.)

**Keywords:** esophageal carcinoma, esophageal adenocarcinoma, esophageal squamous cell carcinoma, epidemiology, risk factors

## Abstract

The two main histological esophageal cancer types, adenocarcinoma and squamous cell carcinoma, differ in incidence, geographic distribution, ethnic pattern and etiology. This article focuses on epidemiology with particular reference to geographic and temporal variations in incidence, along with a review of the evidence supporting environmental and genetic factors involved in esophageal carcinogenesis. Squamous cell carcinoma of the esophagus remains predominantly a disease of the developing world. In contrast, esophageal adenocarcinoma is mainly a disease of western developed societies, associated with obesity and gastro-esophageal reflux disease. There has been a dramatic increase in the incidence of adenocarcinoma in developed countries in parallel with migration of both esophageal and gastric adenocarcinomas towards the gastro-esophageal junction.

## 1. Introduction

Esophageal cancer is the eighth most common cause of cancer death worldwide. There are two main histological types; squamous cell carcinoma (SCC) and adenocarcinoma (ADC). Worldwide, SCC is the predominant histological type. Adenocarcinoma is mainly a disease of developed countries. 

The epidemiology of esophageal cancer differs markedly from other epithelial cancers. There is huge variation in incidence worldwide with greater than 100-fold differences observed between high incidence areas such as China and Iran, and low incidence areas such as Western Africa [1]. These wide variations in incidence are often observed between areas in close geographical proximity [2,3,4]. Male to female incidence rate ratio also varies widely with ratios greater than 20:1 in France to near equality or even excess female cases in high incidence areas such as Iran [2,5]. Worldwide, a higher incidence of esophageal cancer is seen in men with an average 3–4 fold increased rate for SCC and a 7–10 fold increased rate for ADC compared to women [6]. 

This review examines global variation in incidence and temporal trends in epidemiology, and reviews the evidence for the environmental and genetic factors potentially responsible for these differences between SCC and ADC. 

## 2. Methods

MEDLINE and EMBASE were searched independently by two reviewers (RM, OT) for English language articles published between 1972 and February 2010. Search keywords ‘esophageal cancer’, ‘esophageal cancer epidemiology’, ‘esophageal adenocarcinoma epidemiology’, ‘esophageal squamous cell carcinoma epidemiology’, ‘esophageal cancer incidence’, ‘Barrett’s metaplasia’ and ‘Barrett’s esophagus’ were used. All obtained articles were reviewed by both reviewers (RM, OT), with additional review of pertinent references from these publications. Where multiple publications from single institutions were found, only the most recent and relevant articles were included.

Sources of epidemiological data for esophageal cancer including incidence, mortality and survival were ‘Cancer Incidence in Five Continents’ (CI5), volume 9, 2007 and population based registries, including the US Surveillance, Epidemiology and End Results (SEER) registry, GLOBOCAN International Agency for Research on Cancer (IARC) report 2002 and the World Health Organization (WHO) mortality database [7,8]. GLOBOCAN 2002 estimates global incidence of esophageal cancer with publication of total esophageal cancer incidence rates for each country. Where an age-standardized rate (ASR) is included, this is standardized to the world population unless otherwise stated. 

## 3. Results

### 3.1. Epidemiology

**Figure 1:**
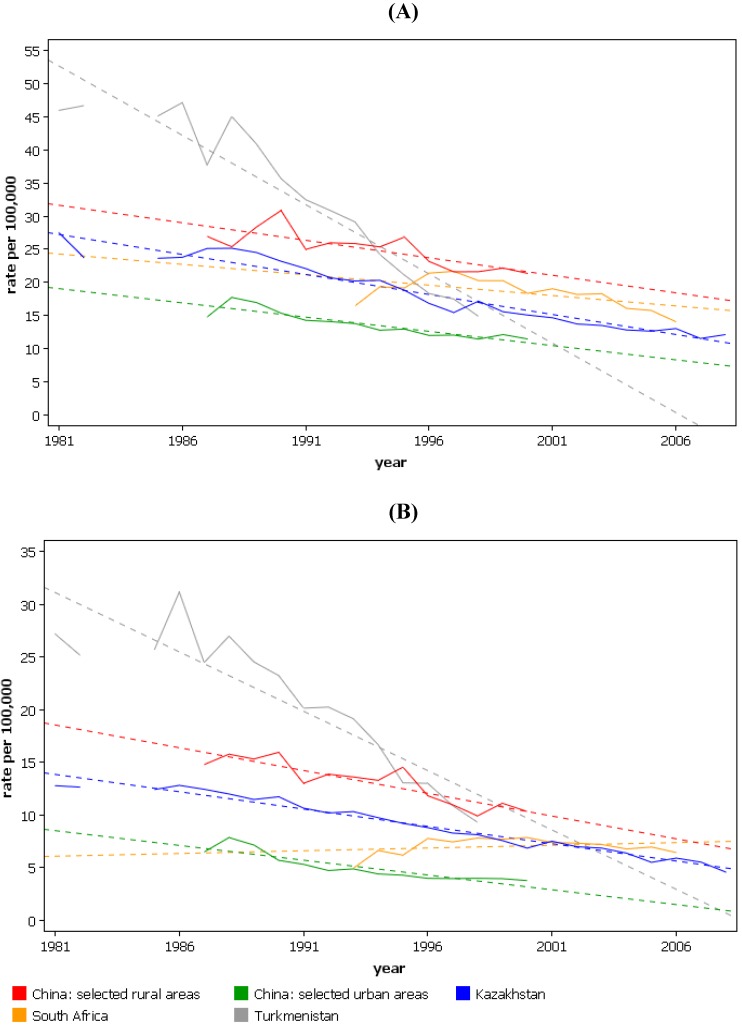
Mortality trends for esophageal cancer in selected countries with highest incidence, age-standardized rates (world). (**A**) males; (**B**) females. (From the WHO mortality database, International Agency for Research on Cancer.)

Countries with the highest incidence of esophageal cancer for males and females are shown in Table 1; trend data for selected high incidence countries is shown in Figure 1. Where the data is available, SCC is the predominant histological type [7].

**Table 1:** Total esophageal cancer rates for the countries with the highest ASR for males and females from the GLOBOCAN 2002 database (ASR are per 100,000 population) [1].

Country	Male
Ethiopia	28.1
China	27.4
Mongolia	24.5
Kenya	22.7
Fiji	21.8
Kazakhstan	20.9
Turkmenistan	20.8
South African Republic	20.8
Tanzania	19.9
Burundi, Comoros, Eritrea, Djibouti, Madagascar, Somalia	19.1
**Country**	**Female**
Mongolia	19.6
Iran, Islamic Republic of	14.4
Turkmenistan	14.1
China	12.0
Sri Lanka	11.8
Uganda, Kazakhstan	11.6
Kenya	11.4
Fiji	11.1
Ethiopia, Qatar	10.1
Malawi	9.9

#### 3.1.1. Squamous cell cancer

In the west, the contribution of SCC to the overall burden of esophageal cancer has decreased due to the rapid rise in the incidence of esophageal ADC. The ASR incidence per 100,000 population observed in the majority of developed western countries is less than 3/100,000 (Table 2) [7]. Despite this, there are geographical areas in the developed world with high rates of SCC in men, including the Calvados region of France (11.8 per 100,000 population) and north-east Italy (7–9 per 100,000 population) [7,9,10]. In addition to regional differences, variations have been observed between ethnic, socioeconomic groups and genders within the same territory. In the US, the incidence of esophageal SCC varies according to ethnicity, race and gender, with rates in males exceeding those in females in all ethnic/racial groups (see Table 4) [11]. During 1977–2005, the SCC rate in black men was four times that of white men (13.6 *vs.* 2.7) [11]. Non-hispanic white males have the lowest incidence (ASR 2.1), American Indians and Alaska Natives the highest (ASR 4.4), and Hispanic (ASR 2.6) and Asian/Pacific Islanders (ASR 3.7) fall in-between (rates are per 100,000 person years, age adjusted to the US 2000 standard population) [11].

Overall, the incidence of SCC is either stable or decreasing in westernized nations, with some exceptions. Increases in SCC were observed in men in Denmark and the Netherlands between 1973 and 2005 [6]. In the US, a decrease has been reported in the incidence of SCC in Blacks, Whites and Hispanics of both sexes; only the rates among Asian/Pacific Islanders did not decrease notably [11]. A significant increase was observed in women in Canada, Scotland, Switzerland and South Australia between 1973 and 2005 [6].

**Figure 2:**
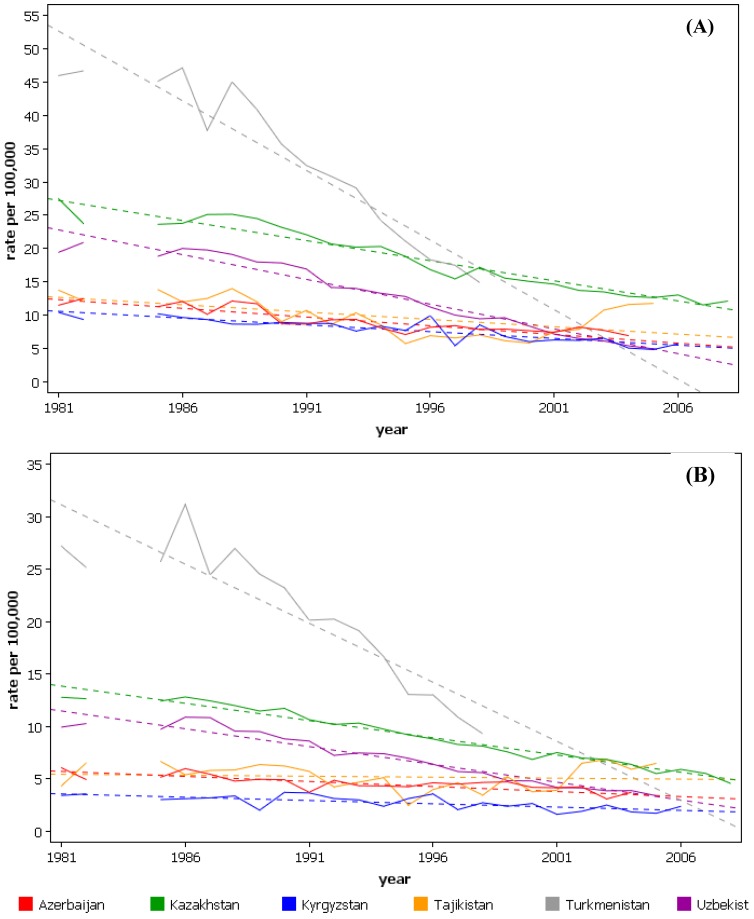
Mortality trends from esophageal cancer for the Central Asian Republics, age-standardized rates (world). (**A**) males; (**B**) females. (From the WHO mortality database, International Agency for Research on Cancer).

Some of the highest rates of esophageal cancer worldwide are seen in the ‘Central Asian esophageal cancer belt’. This region includes the countries of the Caspian littoral, the central Asian republics, Mongolia and north-western China. Although incidence rates by histological type are often unavailable, cancers are almost exclusively squamous enabling overall rates of esophageal cancer to be used as a surrogate of SCC rates [12]. Table 3 shows the incidence of esophageal cancer for the Central Asian republics and Figure 2 shows their esophageal cancer mortality trend data.

**Table 2:** Incidence of esophageal squamous cell carcinoma in westernized countries.

	Country	Male	Female
*North America*			
	US (SEER 14 registries)	1.8	0.8
	Canada	1.5	0.8
*Europe*			
	Denmark	2.5	1.1
	Norway	1.6	0.6
	Sweden	1.5	0.8
	The Netherlands	2.5	1.3
	UK (England)*	1.7–2.7	1.6–2.4
	Scotland	3.9	2.6
	Ireland	2.4	2.1
	France Calvados	11.8	1.3
	Tarn	3.6	0.4
	Italy Veneto	4.6	0.9
	Florence & Prato	1.2	0.3
*Oceania*			
	Australia (South)	0.9	1.0
	New Zealand	1.6	1.1

Age standardized rate incidence per 100,000 population over time period 1998–2002; (* varies by region) [7].

Within high incidence areas there is wide variation in the distribution of disease. In the Caspian littoral, the ASR’s between 1968 and 1970 were reported as 109 and 174 per 100,000 population for men and women, respectively, in Golestan (north-east Iran), compared to less than 20 per 100,000 population in the more westerly province of Gilan [13]. The accuracy of this early study was limited by potential diagnostic errors, with an average of 13% of cases having a histological diagnosis and under-reporting, particularly where medical provision was limited. A similar lower incidence rate of 11.4 and 14.0 per 100,000 person years in males and females, respectively, for the Iranian province of Eastern Azerbaijan on the north-west Caspian Littoral was recorded between 1994 and 2003, with a reduction in incidence rates during the study period. This clinic-based retrospective study was limited by the quality of the demographic data available and may have underestimated incidence rates due to patient diagnosis in neighboring provinces [14]. The incidence of esophageal cancer in the high incidence eastern Caspian Littoral also seems to be in decline with more contemporary reports of ASR’s of 82.6 and 43.4 per 100,000 in men and 95.7 and 36.3 per 100,000 in women [15,16]. The Golestan Cohort Study, a prospective population study which reported ASR’s of 82.6 and 95.7 per 100,000 men and women, respectively, had excellent follow-up with 99.8% four-year follow-up with histological confirmation in all cases [15]. The second retrospective study with reported rates of 43.4 and 36.3 per 100,000 men and women, respectively, may have underestimated rates, with only 68.2% histological confirmation [16]. Some of the variation in incidence within a geographical area may relate to different ethnicities in that area. In Karakalpakstan, the highest incidence reported was in Kazakhs and lowest among Turkmen [4]. The North Indian Kashmir valley that borders the southern Asian esophageal cancer belt is another high risk region, which exhibits variation in incidence over a short distance of 150 km. A prospective study between 1986 and 1989 using histologically proven esophageal cancer reported ASR of 70 per 100,000 men in Islamabad and 17 per 100,000 men in Kupwara. In contrast to other high risk areas (China and Iran), female esophageal cancer rates showed less variability between regions with a greater rate in men compared to women [3]. This study may also have underestimated the true incidence; firstly, not all patients with suspected upper gastrointestinal malignancy have a histological diagnosis, and secondly, in those presenting with metastatic disease, investigation for the primary tumor may not have been undertaken. The Chinese regions of maximum incidence include Cixian in Hebei province and Linxian in Henan [17]. In Cixian the overall incidence of esophageal cancer decreased from 229 to 175 per 100,000 population between 1974 and 1996. This decrease in incidence was most marked in the mountainous regions. Interestingly, an increase was observed over the same period in the dry, plain areas. Furthermore, although a decline was noted in men, the rate in women remained stable [18]. Similar findings were found in Linxian, with declining incidence and mortality rates for esophageal and gastric cardia cancer combined and reduced esophageal cancer rates in Shanghai [19]. Although both these Chinese studies used prospectively collected data, they are subject to inaccuracies arising from changes in diagnostic accuracy, with the potential inclusion of stomach cancers early on, and changes in medical seeking behavior, access to medical services and population mobility.

**Table 3:** Annual esophageal cancer incidence rates per 100,000 population, age adjusted to the world population for the Central Asian Republics of the Central Asian Esophageal Cancer Belt, taken from GLOBOCAN 2002 [1].

Country	Male	Female
Kazakhstan	20.9	11.6
Turkmenistan	20.8	14.1
Azerbaijan	11.5	7.8
Uzbekistan	11.3	6.9
Afghanistan	10.8	8.9
Kyrgyzstan	9.2	3.5
Tajikistan	7.2	6.1

South Africa also harbors a high incidence area in the Transkei region with average annual age standardized (to African standard population) incidence rates of 35.2 and 16.7 per 100,000 population for males and females reported for the period 1965–1969. Within the Transkei, a significant variation in incidence both by geography and ethnicity was reported. The Umzimkulu, Mount Fletcher and Matatiele regions, which are mainly populated by Zulu and Basutu, have low incidence and the Thembu, Fingo and Xhosa areas have higher rates [20]. These figures reflect the total reported cases, of which approximately three quarters were medically confirmed, although the mode of confirmation is not stated.

#### 3.1.2. Adenocarcinoma

Adenocarcinoma has overtaken SCC as the predominant histological type in most western countries. As a result, most cancers in the west occur in the lower esophagus or at the esophago-gastric junction [21,22,23,24]. The worldwide variation in incidence is less marked than with SCC, but similar to SCC, variations in incidence occur between ethnic groups within a country. A key feature of esophageal adenocarcinoma is its male preponderance, contrasting with the near equivalence of male and female incidence rates of SCC in high risk areas. In general, male to female sex ratios in excess of 4:1 are reported throughout North America, The United Kingdom, Australia, Europe and Scandinavia, with the exceptions of Finland and Switzerland, which have male to female ratios of 1.6 and 2.2, respectively [6,7,25,26,27,28,29]. The distribution of ADC differs by race and gender in the US, see Table 4. 

**Table 4:** US esophageal cancer incidence by histologic type, sex and racial/ethnic group (SEER 13 1992–2005).

		Male	Female
*Squamous cell carcinoma*			
White		2.1	1.1
	Non Hispanic	2.1	1.1
	Hispanic	2.6	0.6
Black		9.4	3.3
	American Indian/Alaska Native	4.4	1.1
	Asian/Pacific Islander	3.7	0.8
*Adenocarcinoma*			
White		4.8	0.6
	Non Hispanic	5.0	0.7
	Hispanic	2.8	0.4
Black		1.0	0.3
	American Indian/Alaska Native	2.6	0.8
	Asian/Pacific Islander	0.8	0.2

Rates are per 100,000 person years, age-adjusted using US 2000 standard population. Extracted from [11].

**Figure 3:**
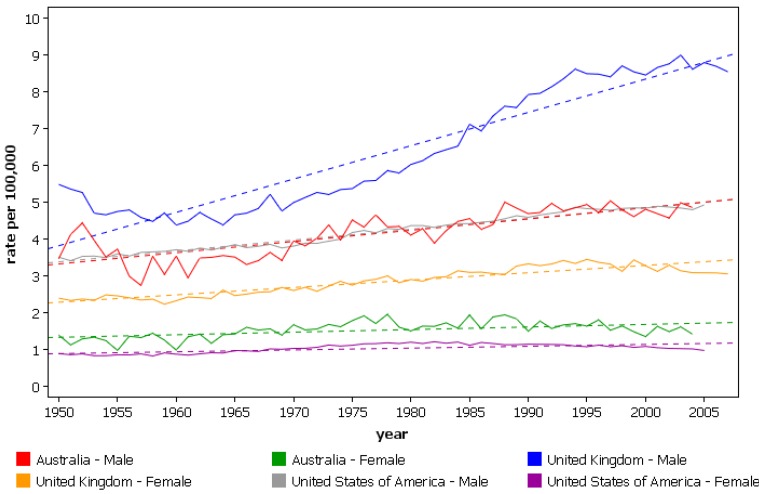
Mortality from esophageal cancer in westernized nations experiencing increased incidence, age-standardized rates (world). (From the WHO mortality database, International Agency for Research on Cancer).

Adenocarcinoma is predominantly a disease of white men; in the US whites are affected five-times more than blacks (3.7 *vs.* 0.8), and men eight-times more than women [11,26]. Marked increases in esophageal cancer due to increased ADC incidence have been observed in the US, Australia, New Zealand and Europe, see Figure 3 [30]. In New Zealand, the increase in incidence is restricted to the non-Maori population [30]. The magnitude of the increase in incidence is large. In the US, increases of 463% (1975–2004, ASR from 1.01 to 5.69/100,000) and 350% (1974–1976 to 1992–1994, ASR from 0.7 to 3.2/100,000) have been reported in white men [22,24]. Similar increases in white females (ASR from 0.1 to 0.4/100,000), and a smaller increase in black males (ASR from 0.4 to 0.6/100,000) between the periods 1974–1976 and 1992–1994 have also been recorded [24]. Increased rates of esophageal ADC have been described in black women and Hispanic men although the rates remained lower than those in white men [11]. The rising incidence is more pronounced in older age groups, with a two-fold increase in incidence seen in men under 65 compared with a 3–4 fold increase in those over the age of 65. A ‘birth cohort effect’ with a 40% increase in incidence for each five-year increase in date of birth has also been observed [21,23,24,26].

The highest incidence of ADC is in the UK with ASR’s (European) in 2006 of 14.1/100,000 men and 5.7/100,000 women (Cancer Research UK). Within the UK, there is geographic variation with the highest incidence in Scotland and lowest in Northern Ireland [7]. A comprehensive study which included all esophageal ADC diagnosed 1971–2001 in England and Wales found an increase in ASR every five years of 39.6% (95% CI 38.6–40.6%) and 37.5% (95% CI 35.8–39.2%) in men and women, respectively. The most rapid rise was amongst the most affluent. A birth cohort effect was seen with the risk for those born in 1940 at 10-times greater risk than the 1900 birth cohort [31]. 

Regional differences in the rate of increase of esophageal ADC incidence have been reported. A six-fold increase in ASR was observed in the West Midlands 1962–1981 [32]. The Merseyside and Cheshire Cancer registry showed an increase in cumulative incidence 1974–1993 from 0.32% to 0.85% in males, and from 0.07% to 0.14% in females. This was almost entirely due to an increase in ADC of the lower esophagus [33]. More modest rises of 4% in men and 17% in women for esophageal ADC are described for the East of England, 1995–2006 [25]. 

In Europe, the reported rates of changing incidence vary. The Eurocim database has been used for many of these studies over different study periods. The inclusion of different regional cancer registries may explain some of these variations. In the Bas Rhin, Calvados, Doubs and Basel regions of France, an increase in the incidence rate per year of 2.8% in men and 5.5% in women has been reported between 1973 and 1992 [6]. However, a similar study between 1968–1995 found no change in incidence but included only two cancer registries (Bas Rhin and Calvados) [34]. There are similar disparate results on the incidence of ADC in Switzerland, with one study reporting an average annual rise of 4.2% in men and 12.1% in women across Basel and Geneva between 1973–1995, while another report found no change in incidence over a longer study period in Basel [6,34]. Investigators agree on increased rates in Scotland, with average annual rises in incidence of 3.1% (men) and 4.8% (women) [6,34]. Botterweck *et al*. also reported an increasing incidence in Varese in Italy, Slovakia and Iceland, with Ireland and the Eindhoven region of The Netherlands showing steady, unchanging rates over a 12 year period to 1995 [34].

In Scandinavia, the picture is much the same. A study from Norway conducted between 1958 and 1992 showed increasing rates of esophageal ADC [28]. Sweden, Denmark and Finland have all experienced similar rises in esophageal ADC incidence [6,27,29]. In Sweden between 1970 and 2004, an average annual increase of 4.9% (ASR from 0.77 to 3.48 per 100,000 population) in men and 3.9% (ASR from 0.21 to 0.66 per 100,000 population) in women has been reported. Of note, the increasing incidence was seen to be accelerating with a 10% increase in men over the latter 15 years of the study [29].

There have also been reports of increasing incidence of esophageal ADC in Japan which has a low prevalence of esophageal ADC compared to the west. An increase in annual death rate from esophageal ADC from 3.7 to 6.9 per 100,000 population has been observed between 1960 and 1995, with a strong association with gastro-esophageal reflux disease (GORD) [35]. 

### 3.2. Etiology

#### 3.2.1. Squamous Carcinoma

Although a number of factors have been implicated, a common etiology and pathogenesis for SCC is yet to be established. One of the most obvious associations is with poverty: with an increasing risk of SCC associated with a decline in socioeconomic status both between and within nations [36,37]. 

##### 3.2.1.1. Diet

Diets lacking fresh fruit and vegetables have been associated with increased esophageal cancer risk in a variety of observational studies conducted in China, France and Iran [36,38,39,40,41]. The incidence of esophageal SCC drops sharply from east to west along the Iranian Caspian littoral from the semi-desert conditions of Golestan towards Gilan which enjoys a subtropical climate, suggesting that climate and the ecosystem, including soil type may be important in the pathogenesis of esophageal SCC [2]. A similar association was noticed in Kazakhstan, where decreased incidence appeared to correlate with increased rainfall [2]. Zinc deficiency has been proposed as a risk factor for esophageal SCC and linked to poor soil conditions in high risk areas of Iran [2]. Zinc deficiency has been shown to enhance carcinogenesis in experimental models of esophageal cancer. Low zinc levels in esophageal biopsies have been correlated with subsequent risk of esophageal SCC in Linxian, China [42]. Selenium status has also been inversely related to SCC risk in China with low serum levels associated with increased risk [43,44]. In the high incidence area of Iran, however, selenium levels were normal [15,45]. 

Hot beverage consumption has been related to increased risk of SCC in South America, Iran, Japan and Singapore [7,46,47,48,49]. However, there is a lack of clarity in the evidence due to difficulties in reliably measuring quantities and temperature of hot drinks consumed. A more recent study carried out in the high risk region of Golestan, Iran, showed increased risk associated with the consumption of hot black tea (odds ratio 2.07, 95% confidence interval 1.28 to 3.35) or very hot black tea (8.16, 3.93 to 16.9) compared to warm or lukewarm black tea. Likewise, increased risk was associated with drinking tea less than three minutes after pouring compared to drinking tea four or more minutes after pouring [50]. Direct and indirect mechanisms have been proposed by which thermal injury may contribute to increased esophageal cancer risk. These include chronic inflammation which may stimulate endogenous formation of reactive nitrogen species and impaired barrier function which may increase the risk of damage by intraluminal carcinogens. Other studies have suggested that carcinogenicity cannot be attributed to the liquid temperature alone and that beverage constituents may play a role. Polycyclic aromatic hydrocarbons (PAH) have been proposed, they are found in mate, a herbal tea consumed in South America which may increase the risk of esophageal cancer even at low temperatures if consumed in sufficient quantities [46,51,52]. Despite these difficulties, hot beverage consumption remains an attractive hypothesis in the etiology of esophageal SCC in some high risk areas such as Golestan because, in contrast with other risk factors such as smoking, opium and alcohol consumption, tea drinking is common with lifelong exposure and is equally distributed between the sexes.

Silica has been proposed as an esophageal carcinogen, with abnormally high levels described in the mucosa of esophageal cancer patients in Northern China [53]. In high incidence areas of SCC, significant silica contamination of common foodstuffs has been described, for example millet bran in Northern China, wheat flour in Iran and porridge made from ‘Cape Chervil’ in The Transkei, South Africa [53,54,55]. A case-control study showed increased esophageal cancer risk with industrial silica exposure in China [53,56]. 

##### 3.2.1.2. Alcohol and Tobacco

The importance of alcohol consumption and smoking in the etiology of esophageal SCC is accepted in western populations. In a US case-control study, the odds ratios for developing esophageal SCC in smokers were 3.1 and 2.5 for white and black males, respectively [37]. Alcohol and tobacco use accounts for the majority of the excess risk of developing SCC in US black males [37,57]. The decline in cigarette smoking by more than a third in US males in the latter part of the twentieth century may have contributed to the observed decline in SCC [24]. Smoking and alcohol consumption play a less significant role in the pathogenesis of esophageal SCC in the high risk areas of the central Asian esophageal cancer belt. In Linxian, a history of smoking was associated with a relative risk of 1.3 of developing SCC and long term smoking conveyed a two-fold risk [41,58]. In Sichuan, odds ratios for the development of esophageal cancer of 4.06 and 2.49 for smoking and alcohol were found [38]. The effect appeared synergistic with an odds ratio of 8.86 for both factors combined [38]. In Golestan, Iran, smoking is associated with a moderately increased risk of esophageal cancer, with a reported prevalence of ever having smoked in the general population of 11%, compared with 25% and 27% of those who developed ADC and SCC respectively reporting a smoking history [12,15]. Alcohol consumption is rare in Golestan and is unlikely to be a major cause of esophageal SCC in thisregion [15,59].

##### 3.2.1.3. Opium and Nass

Opium use is common (estimated 6–14%) in the high risk areas of the Iranian Caspian littoral. Smoking and chewing opium in the form of sukhteh, a cheap alternative that remains in the pipe after smoking, has been shown to be carcinogenic to esophageal mucosa [12,15,60]. A correlation between esophageal cancer risk and urinary opium metabolites has been reported across the Iranian Caspian littoral [60]. Reduced rates of SCC occur alongside decreased opium consumption [15,16,61]. The importance of smoking and opium use in the pathogenesis of esophageal SCC in the high risk areas of China and Iran has to be questioned when one considers the relatively high rates of SCC in women compared to men, where the proportion of women who have ever smoked or used opium is small [15,58]. Nass (a mixture of tobacco and lime) chewing peculiar to Turkmen males in Golestan was postulated to be a risk factor for esophageal SCC. This has not been borne out by epidemiologic studies, which showed low levels of use in the general population (in the Golestan Cohort Study 2008) and among esophageal cancer patients in the Atrak clinic study 2001–2003 [12,15,36].

##### 3.2.1.4. Chemical Carcinogens

Polycyclic aromatic hydrocarbons (PAH) produced by the partial combustion of organic materials have also been implicated. Histopathological studies comparing SCC in Linxian, China with SCC and ADC specimens from the US found increased anthracotic lymph nodes and arteriosclerotic vessels and it was postulated that this may represent histopathological evidence of high level environmental PAH exposure [62]. In the northeast of Iran, two studies have reported urinary levels of a PAH metabolite indicating exposure in greater than 40% of individuals tested, independent of sex, area of residence, smoking, nass or opium use. This paralleled the incidence pattern of SCC [15,63]. *N*-nitroso compounds have been demonstrated to be carcinogenic for esophageal cancer in animal studies [64], but evidence of their importance in the etiology of SCC from epidemiologic studies is conflicting [65,66].

##### 3.2.1.5. Chronic Inflammation

High rates of chronic non-erosive esophagitis have been described in China, Iran and the Transkei areas with high rates of SCC. In China, 65% of males and 63.5% of females from a high risk population undergoing endoscopy had biopsy-proven chronic esophagitis [67]. In a similar study in northern Iran, chronic esophagitis was seen in 80%, with a very high incidence even in young patients [68]. In the Transkei, 24% of adults from a high risk area had esophagitis detected by esophageal cytology smears; significantly greater than in an adjacent low risk area [69]. The chronic non-erosive esophagitis observed in both Iran and China was histologically distinct from the erosive esophagitis seen in western populations associated with GORD. Chronic non-erosive esophagitis has therefore been considered a precursor to invasive SCC. 

A recent study carried out in the same high risk region of China, followed a cohort of patients with cytological diagnoses of normal esophageal epithelium, esophagitis, esophageal dysplasia, or carcinoma *in situ* over 13.5 years. Esophagitis conferred no greater risk of invasive SCC than those with normal esophageal epithelium. The risk of subsequent esophageal cancer increased with the degree of dysplasia, with high grade dysplasia and carcinoma *in situ* having a similar risk [70]. This study brings into question the role of chronic inflammation in the pathogenesis of esophageal SCC.

In the west, chronic esophagitis usually results from GORD, and may be erosive or non-erosive. The risk of erosive esophagitis in patients with reflux symptoms is increased by smoking and alcohol consumption [71]. Despite an endoscopic survey in the high risk area of Normandy that showed a prevalence of histologically-proven chronic esophagitis of 63%, there was no convincing evidence that this increased the risk of SCC [72].

Achalasia has been associated with an increased risk of esophageal SCC, but the magnitude of risk is uncertain. In a cohort of 124 achalasia patients undergoing 1–2 yearly endoscopy for an average of 5.6 years in a single institution from 1982, the risk of developing SCC was 140-times the general population [73]. A more recent study, which included 226 patients post cardiomyotomy and partial fundoplication with 18.3 years average follow-up, found an increased risk of SCC only in men with a standardized mortality ratio of 11.01 [74]. Chronic inflammation caused by the retention of fermenting food debris has been proposed as the likely mechanism. 

Caustic injury causes intense inflammation and stricturing, and increases the risk of esophageal SCC approximately 1000-fold [75,76]. The time from ingestion to presentation varies, but an interval of 20–40 years is common. Because accidental ingestion often occurs in children, the resulting tumors tend to be observed in a younger age group than other esophageal cancers [76,77].

##### 3.2.1.6. Genetic Susceptibility

The epidemiologic evidence presented shows environmental risk factors for SCC. However, only a subset of exposed individuals will develop SCC, suggesting that genetic factors may be involved in its pathogenesis. The ‘Asian esophageal cancer belt’ largely corresponds with trade routes, especially the Silk route. It has been proposed that the spread of environmental or lifestyle risk factors common to the Turkic tribes and Mongol conquerors who traded and settled this region may be important. Early epidemiologic studies suggested increased susceptibility amongst Turkmen compared to Persians in the high risk Caspian littoral of Iran and proposed that the high incidence of esophageal SCC along the trade route may be due to high penetrance of susceptibility genes spread by Turkmen and similar groups, who had a nomadic lifestyle and migrated from East Asia and inhabit much of the Silk Route, possibly as a result of trading activity. A case control study performed in northeast Iran compared the pedigrees of Turkmen SCC patients with the pedigrees of matched Turkmen controls. They demonstrated that the risk to age 75 of esophageal cancer in the first-degree relatives of Turkmen patients with esophageal cancer was 34% *versus* 14% for the first-degree relatives of controls (hazard ratio: 2.3). The authors concluded that the findings were consistent with an important contribution of heritable factors to the pathogenesis of esophageal cancer in this population [78]. A study reported in 2004 suggested that the risk of developing esophageal SCC in north-east Iran was the same among Turkmen and non-Turkmen [12]. 

Although a number of genetic abnormalities have been associated with esophageal cancer, these vary between high risk populations and no single gene has been identified as pivotal in the pathogenesis of esophageal cancer. Genetic polymorphisms in STK15 and MMP-2 have been associated with increased risk of esophageal cancer in Mongolia. However, their frequency was significantly different in a high risk Han Chinese population suggesting differences in genetic susceptibility to esophageal cancer between high risk regions [79]. 

Alcohol dehydrogenases (ADH) oxidize ethanol to acetaldehyde, which is in turn oxidized to aldehyde by aldehyde dehydrogenase (ALDH). Acetaldehyde has been demonstrated to be carcinogenic and therefore genetic variations resulting in functional differences in ADH and ALDH activity which lead to increased levels of acetaldehyde in drinkers may be important [80]. A number of the ADH and ALDH families have been implicated in the pathogenesis of SCC [81]. The ability to metabolize acetaldehyde is encoded by the ALDH2 gene with the ALDH2*2 allele producing an inactive protein, unable to metabolize acetaldehyde. Interestingly, compared to ALDH2*1*1 homozygotes individuals who are ALDH2*2*2 homozygotes are protected from SCC whilst ALDH2*1*2 heterozygotes have increased risk. In ALDH2*1*2 heterozygotes the increased risk was modified by alcohol consumption with no appreciable increase in risk among non-drinkers, moderately increased risk in moderate drinkers (OR 2.49; 95% CI 1.39–4.49) and the highest risk (OR 7; 95% CI 3.07–13.6) in heavy drinkers [82,83]. It has been proposed that genotype at the ALDH locus may influence SCC risk by two mechanisms, by modifying alcohol intake and by influencing acetaldehyde levels and therefore that the protective effect of the inactive genotype ALDH2*2*2 may be mediated through lower alcohol intake because of the adverse facial flushing they experience on alcohol consumption due to acetaldehyde accumulation. The genotype ALDH2*504Lys also functions as a dominant negative, reducing activity in heterozygotes and abolishing activity in homozygotes. Its distribution has been mapped globally, and is restricted to East Asia with the highest frequency occurring in southern China with frequencies declining radially through China, Japan, Korea, Mongolia and Indochina. Although its distribution does not coincide with the area of highest incidence in China, it has been suggested that the genotype was carried by Han Chinese as they spread throughout East Asia. Of interest, low frequencies of ALDH2*504Lys are also seen in Kazakhstan and Tajikistan, areas of moderate to high esophageal cancer incidence [1,84]. However, this hypothesis does not explain the dominant contribution of alcohol to the risk of esophageal SCC in Western populations where the inactive ALDH allele is not observed [84]. 

A comprehensive Japanese two-step genome wide association study including 1070 SCC cases and 2836 controls identified significant associations of SCC with single nucleotide polymorphisms (SNP) in ADH1B and ALDH2 with odds rations of 1.85 (95% CI 1.03–3.34) and 1.66 (1.11–2.50) respectively. They went on to perform logistic regression analysis to estimate gene-gene and gene-environmental interactions and found that increased risk in ALDH2 polymorphism was increased to a greater extent amongst smokers, those with heavy alcohol consumption and in the young population, whereas risk with ADH1B polymorphism was accentuated only with alcohol. Polymorphisms at both loci acted synergistically with an OR for SCC *versus* a no risk genotype of 16.17 (11.55–22.65); individuals with both genetic polymorphisms who smoked and had heavy alcohol consumption exhibited a 190-times higher risk than those who had neither [85]. Acetaldehyde is a component of cigarette smoke which may explain the synergistic effect of smoking and ALDH2 mutation on SCC risk.

Polymorphic variation in the DNA repair capacity genes XPD and MGMT as well as ALD2 have been associated with increased esophageal SCC susceptibility in a Chinese population study [86]. Xenobiotics are thought to be activated to carcinogens by phase 1 enzymes and subsequently detoxified by phase 2 enzymes such as cytochrome p450 1A1 (CYT1A1) and glutathione *S*-transferase M1 (GSTM1) and that decreased phase 2 enzymes may increase carcinogen activity [87]. Another study investigated the incidence of the null genotype (homozygous deletions resulting in absent enzyme activity) of glutathione-S-transferase M1 (GSTM1) in three Chinese minorities, Kazakh, Uygur, and Tajik that experience varying incidence of esophageal SCC in a high incidence area. They found a high frequency in GSTM1 null genotype in all three ethnic groups (47.4–62.63%) with differing frequencies between the groups and an association with poorly differentiated SCC. However, a recent meta-analysis investigating GSTM1 and CYP1A1 polymorphisms found that CYP1A1 exon 7 polymorphism was associated with increased risk in Asians only but that GSTM1 polymorphism was not associated with SCC risk [87].

Recently, interest has turned to microRNAs (miRNA). These are a class of small non-coding RNAs that function as post-transcriptional regulators of gene expression which are involved in cell differentiation, proliferation and apoptosis and may function as oncogenes and tumor suppressor genes [88]. Aberrant miRNA expression involving a whole host of specific miRNAs has been identified in esophageal SCC and ADC, with each having a different miRNA profile in keeping with different etiologic characteristics and potentially involved in their carcinogenesis [89]. RNA-specific endonucleases (RNASEN) are involved in cleaving precursor miRNA in a stepwise fashion to generate mature miRNA and have been implicated in the development of SCC with amplification of RNASEN reported in SCC specimens and growth suppression of SCC cell lines with RNASEN inhibition [90].

#### 3.2.2. Adenocarcinoma

As well as the artifactual change due to improvements in histological verification with less cases of esophageal cancer being classified ‘unspecified’ and changes in the classification of tumors at the gastro-esophageal junction, the widespread increase in incidence appears to be a real and sustained phenomenon. The etiology of esophageal adenocarcinoma is strongly linked to gastro-esophageal reflux disease (GORD), Barrett’s esophagus and obesity. Smoking, although less important than in the etiology of SCC in the west, appears to increase the risk of ADC, possibly by reducing lower esophageal sphincter pressure and promoting reflux [91].

##### 3.2.2.1. GORD

Gastro-esophageal reflux disease is an important risk factor for ADC. The reported strength of association between GORD and ADC varies [92]. Three UK studies have estimated the risk of developing esophageal ADC in patients with GORD, esophagitis or Barrett’s esophagus and found relative risks (RR) of 30 for Barrett’s; 4.5 and 6.9 for esophagitis; and 3 and 1.67 for GORD compared to reference cohorts [93,94,95]. Interestingly, an increase in GORD has been reported in US veterans, and if widespread, may have contributed to the increased incidence of ADC [96].

##### 3.2.2.2. Barrett’s Metaplasia

It is widely accepted that esophageal adenocarcinoma arises predominantly in Barrett’s esophagus consequent to reflux [97]. Factors involved in the transition from esophagitis to columnar (Barrett’s) epithelium involve acid and bile reflux facilitated by a weak lower esophageal sphincter with increased transient relaxations, compromised esophageal clearance and esophageal dysmotility [17]. The presence of intestinal metaplasia (IM) within the columnar segment appears important in the progression to neoplasia. Intestinal metaplasia is probably an adaptive phenomenon, with mucous production by goblet cells increasing mucosal defences to noxious refluxate. Why this remains stable in some patients and becomes unstable in others remains unknown. Duodeno-gastro-esophageal reflux including bile salts has been implicated in the pathogenesis of Barrett’s esophagus and progression to ADC. The increasing use of acid suppressive drugs which raise the pH of the refluxate has been suggested as a contributor to the increasing incidence of Barrett’s esophagus and ADC [6,98]. Experimental animal studies show that gastro-duodenal reflux causes esophageal cancer, of which the majority are adenocarcinoma [99]. 

Much attention has been focused on the declining prevalence of *H. pylori* infection as the incidence of esophageal ADC has increased and there are conflicting reports regarding the relationship between *H. pylori* infection and risk of esophageal ADC. A number of studies have found that infection with cagA+ *H.pylori* strains is associated with a decreased risk of esophageal and cardia ADC [100,101]. However, a population based study in Los Angeles found no evidence that CagA+ strains of *H.pylori* reduce the risk of esophageal ADC [102]. More studies are warranted to establish the potential inverse relation between *H. pylori* and the risk of esophageal adenocarcinoma. *H. pylori* infection is characterized by either pan-gastritis with hypochlorhydria or antral gastritis with acid hypersecretion. The host inflammatory response modulated by pro-inflammatory polymorphisms in IL-1β and TNFα are two factors that have been found to distinguish between subjects who will develop the hypochlorhydric atrophic phenotype in response to *H pylori* and those who manage to limit the infection to a smaller area and offer better protection of their corpus function [103]. This may help to explain the differing results of studies examining the effect of *H. pylori* infection on esophageal ADC. It has been proposed that pan-gastritis with atrophy may be associated with decreased risk of ADC due to hypochlorhydria with less acid available to damage the esophageal mucosa.

Reports of the rate of progression from Barrett’s metaplasia to invasive cancer vary with the population studied between 1 per 100 and 1 per 200 patient years, with the risk of esophageal ADC increased 30-fold by the presence of Barrett’s [104,105,106]. Risk of progression is increased by male sex, current smoking, obesity, the presence of long segment Barrett’s and ulceration. There is evidence that the risk of developing adenocarcinoma in Barrett’s is declining. This may be due to differences in patients being recruited to and adhering to surveillance programmes. Other possible explanations for the declining rate of ADC in Barrett’s may be effective treatment with anti-reflux therapy, or the use of NSAIDs and aspirin which have been associated with regression of Barrett’s and reduced risk of esophageal cancer respectively [107]. In terms of explaining the rapid increase in the incidence of esophageal ADC, an increase in Barrett’s esophagus of 41% in men and 23% in women from 1996–1999 that was not due to changes in endoscopic practice or histopathologic criteria has been reported in a Dutch study [108].

##### 3.2.2.3. Obesity

The increased incidence of obesity has paralleled that of esophageal ADC with studies from the US demonstrating this between 1990–2000 by age and sex from SEER data and an increase in overweight individuals by 8% between the periods 1976–1980 and 1988–1991 [109,110]. Subsequently, obesity has been investigated as a potential etiological factor. It has been proposed that obesity might increase the risk of esophageal ADC by increasing intra-abdominal pressure and predisposing to gastro-esophageal reflux. A number of case control studies have investigated the effect of obesity on the risk of esophageal ADC, and have found that the highest risk was associated with individuals with a body mass index (BMI) in the top 10% (OR 2.5 95% CI 1.2–5.0) or in the highest *vs.* the lowest quartile of BMI (OR 3.1, CI 1.8–5.3) [111,112]. A third study had similar findings, reporting a dose dependent relationship, with increasing risk of esophageal ADC seen with increasing BMI (BMI > 25 kg/m^2^: OR 1.67 (95% CI 1.22–2.3) and BMI > 35 kg/m^2^: OR 3.68 (95% CI 1.81–7.51)). This effect was independent of gastro-esophageal reflux, suggesting that the risk of esophageal ADC in obesity may not be mediated through excess reflux as has been proposed [113]. However, the severity-dependent risk factors for esophageal ADC, obesity and GORD seem to increase the risk in a multiplicative way, with the greatest risk observed with obesity and reflux disease (adjusted odds ratio 179.2) [114]. A prospective nested case control study carried out in the UK between 1994 and 2001 found that the effect of BMI on esophageal ADC risk varied with sex. Interestingly, increased risk of esophageal ADC was restricted in women to those who were obese with a BMI > 30 kg/m^2^ whilst in men the risk of esophageal ADC started to climb at more modest weight increases with a BMI > 25 kg/m^2^ [113]. 

Subsequent to the reports of obesity as a risk factor for esophageal ADC, its effect on the development of Barrett’s esophagus has attracted interest. A meta-analysis including nine studies that compared patients with Barrett’s esophagus with GORD patients found no difference in their BMIs (OR 0.99 per kg/m^2^; 95% CI 0.97–1.01), while the pooled estimate of three studies comparing Barrett’s esophagus with general population controls was 1.02 per kg/m^2^ (95% CI 1.01–1.04). They concluded that obesity was an indirect risk factor for Barrett’s esophagus and mediated its effect through its precursor lesion, GORD [115]. Of interest, an inverse association between the anti-inflammatory, low molecular weight form of adiponectin and Barrett’s esophagus has been noted among patients with GORD [116]. Furthermore, abdominal (male pattern) body fat has been identified as a strong predictor of progression of Barrett’s esophagus to ADC [117]. This may help to explain the high male to female ratio of ADC and the increased risk of progression of Barrett’s esophagus in males. 

##### 3.2.2.4. Genetic Susceptibility

A genetic susceptibility to esophageal ADC was suspected when familial clustering of esophageal ADC and Barrett’s esophagus were reported [81,118]. Furthermore, Barrett’s esophagus has been confirmed in first or second degree relatives of 7.3% of patients presenting with Barrett’s esophagus, esophageal or gastro-esophageal junction ADC [119]. A number of candidate genes have been proposed including polymorphisms in enzymes involved in the detoxification of xenobiotics, but none have been proven to increase risk of Barrett’s esophagus or ADC and further studies are required [81]. MicroRNAs have also been implicated in the pathogenesis of ADC with different miRNA expression profiles in normal squamous epithelium, Barrett’s metaplasia, Barrett’s metaplasia with high grade dysplasia, ADC and SCC [89,120,121]. Of particular importance is the identification of different miRNA profiles between Barrett’s esophagus with high grade dysplasia and normal squamous epithelium or Barrett’s with low grade dysplasia which may be important in disease progression to ADC and may be markers of tumor progression which may have potential in developing biomarkers for identifying patients with Barrett’s esophagus who are at high risk of progression to ADC to be selected for aggressive treatment [120].

## 4. Summary

In summary, the epidemiology of esophageal cancer is changing. Quantifying the contribution of environmental and genetic factors to esophageal cancer risk is limited by the quality of data from many countries. In the west, there has been a dramatic increase in the incidence of esophageal ADC with a less marked but consistent reduction in the incidence of squamous cell carcinoma. The duration and final magnitude of the increase in incidence of ADC remains speculative but there is some evidence that it may be slowing down. One study from the UK examining incidence trends of ADC to 2006 demonstrated a possible levelling off in the rate of increase [25]. The wide variation in incidence of ADC between countries remains unexplained. However, the increase in ADC appears to be due, at least in part to the increase in obesity which increases the risk of Barrett’s esophagus through increased GORD. Furthermore, central obesity may promote the progression of Barrett’s esophagus to adenocarcinoma although more work is needed. The decrease in SCC observed throughout almost all high risk regions, as well as in westernized nations may be attributable to declines in smoking and moderate to high alcohol consumption and dietary improvements with increased intake of fresh fruit and vegetables alongside improvements in socioeconomic status and poverty. Understanding the genetic changes that are specific to groups which are defined by ethnicity that render them susceptible to environmental carcinogens which may also be geographically or culturally restricted may shed light on the etiology and geographic variation of both SCC and ADC.

## Abbreviations

Adenocarcinoma: ADC
Gastro-esophageal reflux disease: GORD
International Agency for Research on Cancer: IARC
Intestinal metaplasia: IM
Polycyclic aromatic hydrocarbons: PAH
Surveillance, Epidemiology and End Results: SEER
Squamous cell carcinoma: SCC
World Health Organization: WHO

## References

[1] Ferlay J., Bray F., Pisani P., Parkin D.M. (2004). GLOBOCAN 2002: Cancer Incidence, Mortality and Prevalence Worldwide.

[2] Kmet J., Mahboubi E. (1972). Esophageal cancer in the Caspian littoral of Iran: initial studies. Science.

[3] Khuroo M.S., Zargar S.A., Mahajan R., Banday M.A. (1992). High incidence of esophageal and gastric cancer in Kashmir in a population with special personal and dietary habits. Gut.

[4] Zaridze D.G., Basieva T., Kabulov M., Day N.E., Duffy S.W. (1992). Esophageal cancer in the Republic of Karakalpakstan. Int. J. Epidemiol..

[5] Day N.E. (1975). Some aspects of the epidemiology of esophageal cancer. Cancer Res..

[6] Vizcaino A.P., Moreno V., Lambert R., Parkin D.M. (2002). Time trends incidence of both major histologic types of esophageal carcinomas in selected countries, 1973–1995. Int. J. Cancer.

[7] Curado M.P., Edwards B., Shin H.R., Storm H., Ferlay J., Heanue M., Boyle P. (2007). Cancer Incidence in Five Continents, IARC Sci. Publ. No. 160.

[8] Horner M.J., Ries L., Krapcho M., Neyman N., Aminou R., Howlader N., Altekruse S.F., Feuer E.J., Huang L., Mariotto A., Miller B.A., Lewis D.R., Eisner M.P., Stinchcomb D.G., Edwards B.K. (2009). SEER Cancer Statistics Review, 1975–2006.

[9] Zambon P., Talamini R., Vecchia C.L., Maso L.D., Negri E., Tognazzo S., Simonato L., Franceschi S. (2000). Smoking, type of alcoholic beverage and squamous-cell esophageal cancer in northern Italy. Int. J. Cancer.

[10] Negri E., La Vecchia C., Franceschi S., Decarli A., Bruzzi P. (1992). Attributable risks for esophageal cancer in northern Italy. Eur. J. Cancer.

[11] Cook M.B., Chow W.H., Devesa S.S. (2009). Esophageal cancer incidence in the United States by race, sex, and histologic type, 1977–2005. Br. J. Cancer.

[12] Islami F., Kamangar F., Aghcheli K., Fahimi S., Semnani S., Taghavi N., Marjani H.A., Merat S., Nasseri-Moghaddam S., Pourshams A. (2004). Epidemiologic features of upper gastrointestinal tract cancers in Northeastern Iran. Br. J. Cancer.

[13] Mahboubi E., Kmet J., Cook P.J., Day N.E., Ghadirian P., Salmasizadeh S. (1973). Esophageal cancer studies in the Caspian Littoral of Iran: the Caspian cancer registry. Br. J. Cancer.

[14] Gholipour C., Shalchi R.A., Abbasi M. (2008). A histopathological study of esophageal cancer on the western side of the Caspian littoral from 1994 to 2003. Dis. Esophagus.

[15] Pourshams A., Khademi H., Malekshah A.F., Islami F., Nouraei M., Sadjadi A.R., Jafari E., Rakhshani N., Salahi R., Semnani S. (2009). Cohort Profile: The Golestan Cohort Study—a prospective study of esophageal cancer in northern Iran. Int. J. Epidemiol..

[16] Semnani S., Sadjadi A., Fahimi S., Nouraie M., Naeimi M., Kabir J., Fakheri H., Saadatnia H., Ghavamnasiri M.R., Malekzadeh R. (2006). Declining incidence of esophageal cancer in the Turkmen Plain, eastern part of the Caspian Littoral of Iran: a retrospective cancer surveillance. Cancer Detect. Prev..

[17] Lambert R., Hainaut P. (2007). Epidemiology of esophagogastric cancer. Best Pract. Res. Clin. Gastroenterol..

[18] He Y.T., Hou J., Qiao C.Y., Chen Z.F., Song G.H., Li S.S., Meng F.S., Jin H.X., Chen C. (2003). An analysis of esophageal cancer incidence in Cixian county from 1974 to 1996. World J. Gastroenterol..

[19] Ke L. (2002). Mortality and incidence trends from esophagus cancer in selected geographic areas of China circa 1970–90. Int. J. Cancer.

[20] Rose E.F., McGlashan N.D. (1975). The spatial distribution of esophageal carcinoma in the Transkei, South Africa. Br. J. Cancer.

[21] Zheng T., Mayne S.T., Holford T.R., Boyle P., Liu W., Chen Y., Mador M., Flannery J. (1992). Time trend and age-period-cohort effects on incidence of esophageal cancer in Connecticut, 1935-89. Cancer Causes Contr..

[22] Brown L.M., Devesa S.S., Chow W.H. (2008). Incidence of adenocarcinoma of the esophagus among white Americans by sex, stage, and age. J. Natl. Cancer Inst..

[23] Crane S.J., Richard Locke G., Harmsen W.S., Diehl N.N., Zinsmeister A.R., Joseph Melton L., Romero Y., Talley N.J. (2007). The changing incidence of esophageal and gastric adenocarcinoma by anatomic sub-site. Aliment. Pharmacol. Ther..

[24] Devesa S.S., Blot W.J., Fraumeni J.F. (1998). Changing patterns in the incidence of esophageal and gastric carcinoma in the United States. Cancer.

[25] Gajperia C., Barbiere J.M., Greenberg D., Wright K., Lyratzopoulos G. (2009). Recent incidence trends and sociodemographic features of esophageal and gastric cancer types in an English region. Aliment. Pharmacol. Ther..

[26] El-Serag H.B., Mason A.C., Petersen N., Key C.R. (2002). Epidemiological differences between adenocarcinoma of the esophagus and adenocarcinoma of the gastric cardia in the USA. Gut.

[27] Sihvo E.I., Salminen J.T., Ramo O.J., Salo J.A. (2000). The epidemiology of esophageal adenocarcinoma: has the cancer of gastric cardia an influence on the rising incidence of esophageal adenocarcinoma?. Scand. J. Gastroenterol..

[28] Hansen S., Wiig J.N., Giercksky K.E., Tretli S. (1997). Esophageal and gastric carcinoma in Norway 1958–1992: Incidence time trend variability according to morphological subtypes and organ subsites. Int. J. Cancer.

[29] Falk J., Carstens H., Lundell L., Albertsson M. (2007). Incidence of carcinoma of the esophagus and gastric cardia. Changes over time and geographical differences. Acta Oncol..

[30] Armstrong R.W., Borman B. (1996). Trends in Incidence Rates of Adenocarcinoma of the Esophagus and Gastric Cardia in New Zealand, 1978–1992. Int. J. Epidemiol..

[31] Lepage C., Rachet B., Jooste V., Faivre J., Coleman M.P. (2008). Continuing rapid increase in esophageal adenocarcinoma in England and Wales. Am. J. Gastroenterol..

[32] Macfarlane G.J., Boyle P. (1994). The epidemiology of esophageal cancer in the UK and other European countries. J. R. Soc. Med..

[33] Dolan K., Sutton R., Walker S.J., Morris A.I., Campbell F., Williams E.M. (1999). New classification of esophageal and gastric carcinomas derived from changing patterns in epidemiology. Br. J. Cancer.

[34] Botterweck A.A., Schouten L.J., Volovics A., Dorant E., van den Brandt P.A. (2000). Trends in incidence of adenocarcinoma of the esophagus and gastric cardia in ten European countries. Int. J. Epidemiol..

[35] Hongo M. (2004). Review article: Barrett's esophagus and carcinoma in Japan. Aliment. Pharmacol. Ther..

[36] Cook-Mozaffari P.J., Azordegan F., Day N.E., Ressicaud A., Sabai C., Aramesh B. (1979). Esophageal cancer studies in the Caspian Littoral of Iran: results of a case-control study. Br. J. Cancer.

[37] Brown L.M., Hoover R., Silverman D., Baris D., Hayes R., Swanson G.M., Schoenberg J., Greenberg R., Liff J., Schwartz A. (2001). Excess incidence of squamous cell esophageal cancer among US Black men: role of social class and other risk factors. Am. J. Epidemiol..

[38] Yang C.X., Wang H.Y., Wang Z.M., Du H.Z., Tao D.M., Mu X.Y., Chen H.G., Lei Y., Matsuo K., Tajima K. (2005). Risk factors for esophageal cancer: a case-control study in South-western China. Asian Pac. J. Cancer Prev..

[39] Launoy G., Milan C., Day N.E., Pienkowski M.P., Gignoux M., Faivre J. (1998). Diet and squamous-cell cancer of the esophagus: a French multicentre case-control study. Int. J. Cancer.

[40] Hormozdiari H., Day N.E., Aramesh B., Mahboubi E. (1975). Dietary factors and esophageal cancer in the Caspian Littoral of Iran. Cancer Res..

[41] Guo W., Blot W.J., Li J.Y., Taylor P.R., Liu B.Q., Wang W., Wu Y.P., Zheng W., Dawsey S.M., Li B. (1994). A nested case-control study of esophageal and stomach cancers in the Linxian nutrition intervention trial. Int. J. Epidemiol..

[42] Abnet C.C., Lai B., Qiao Y.L., Vogt S., Luo X.M., Taylor P.R., Dong Z.W., Mark S.D., Dawsey S.M. (2005). Zinc concentration in esophageal biopsy specimens measured by x-ray fluorescence and esophageal cancer risk. J. Natl. Cancer Inst..

[43] Wei W.Q., Abnet C.C., Qiao Y.L., Dawsey S.M., Dong Z.W., Sun X.D., Fan J.H., Gunter E.W., Taylor P.R., Mark S.D. (2004). Prospective study of serum selenium concentrations and esophageal and gastric cardia cancer, heart disease, stroke, and total death. Am. J. Clin. Nutr..

[44] Mark S.D., Qiao Y.L., Dawsey S.M., Wu Y.P., Katki H., Gunter E.W., Fraumeni J.F., Blot W.J., Dong Z.W., Taylor P.R. (2000). Prospective study of serum selenium levels and incident esophageal and gastric cancers. J. Natl. Cancer Inst..

[45] Nouarie M., Pourshams A., Kamangar F., Sotoudeh M., Derakhshan M.H., Akbari M.R., Fakheri H., Zahedi M.J., Caldwell K., Abnet C.C. (2004). Ecologic study of serum selenium and upper gastrointestinal cancers in Iran. World J. Gastroenterol..

[46] Castellsague X., Munoz N., De Stefani E., Victora C.G., Castelletto R., Rolon P.A. (2000). Influence of mate drinking, hot beverages and diet on esophageal cancer risk in South America. Int. J. Cancer.

[47] De Jong U.W., Breslow N., Hong J.G., Sridharan M., Shanmugaratnam K. (1974). Aetiological factors in esophageal cancer in Singapore Chinese. Int. J. Cancer.

[48] Ghadirian P. (1987). Thermal irritation and esophageal cancer in northern Iran. Cancer.

[49] Segi M. (1975). Tea-gruel as a possible factor for cancer for the esophagus. Gann.

[50] Islami F., Pourshams A., Nasrollahzadeh D., Kamangar F., Fahimi S., Shakeri R., Abedi-Ardekani B., Merat S., Vahedi H., Semnani S. (2009). Tea drinking habits and esophageal cancer in a high risk area in northern Iran: population based case-control study. BMJ.

[51] Sewram V., De Stefani E., Brennan P., Boffetta P. (2003). Mate consumption and the risk of squamous cell esophageal cancer in uruguay. Cancer Epidemiol. Biomarkers Prev..

[52] Fagundes R.B., Abnet C.C., Strickland P.T., Kamangar F., Roth M.J., Taylor P.R., Dawsey S.M. (2006). Higher urine 1-hydroxy pyrene glucuronide (1-OHPG) is associated with tobacco smoke exposure and drinking mate in healthy subjects from Rio Grande do Sul, Brazil. BMC Cancer.

[53] O'Neill C., Pan Q., Clarke G., Liu F., Hodges G., Ge M., Jordan P., Chang U., Newman R., Toulson E. (1982). Silica fragments from millet bran in mucosa surrounding esophageal tumours in patients in northern China. Lancet.

[54] O'Neill C.H., Hodges G.M., Riddle P.N., Jordan P.W., Newman R.H., Flood R.J., Toulson E.C. (1980). A fine fibrous silica contaminant of flour in the high esophageal cancer area of north-east Iran. Int. J. Cancer.

[55] Parry D.W., O'Neill C.H., Hodson M.J. (1986). Opaline Silica Deposits in the Leaves of Bidens pilosa L. and their Possible Significance in Cancer. Ann. Bot..

[56] Pan G., Takahashi K., Feng Y., Liu L., Liu T., Zhang S., Liu N., Okubo T., Goldsmith D.F. (1999). Nested case-control study of esophageal cancer in relation to occupational exposure to silica and other dusts. Am. J. Ind. Med..

[57] Brown L.M., Hoover R.N., Greenberg R.S., Schoenberg J.B., Schwartz A.G., Swanson G.M., Liff J.M., Silverman D.T., Hayes R.B., Pottern L.M. (1994). Are racial differences in squamous cell esophageal cancer explained by alcohol and tobacco use?. J. Natl. Cancer Inst..

[58] Tran G.D., Sun X.-D., Abnet C.C., Fan J.-H., Dawsey S.M., Dong Z.-W., Mark S.D., Qiao Y.-L., Taylor P.R. (2005). Prospective study of risk factors for esophageal and gastric cancers in the Linxian general population trial cohort in China. Int. J. Cancer.

[59] Pourshams A., Saadatian-Elahi M., Nouraie M., Malekshah A.F., Rakhshani N., Salahi R., Yoonessi A., Semnani S., Islami F., Sotoudeh M. (2005). Golestan cohort study of esophageal cancer: feasibility and first results. Br. J. Cancer.

[60] Ghadirian P., Stein G.F., Gorodetzky C., Roberfroid M.B., Mahon G.A., Bartsch H., Day N.E. (1985). Esophageal cancer studies in the Caspian littoral of Iran: some residual results, including opium use as a risk factor. Int. J. Cancer.

[61] Kamangar F., Malekzadeh R., Dawsey S.M., Saidi F. (2007). Esophageal cancer in Northeastern Iran: a review. Arch. Iran Med..

[62] Roth M.J., Guo-Qing W., Lewin K.J., Ning L., Dawsey S.M., Wesley M.N., Giffen C., Yong-Qiang X., Maher M.M., Taylor P.R. (1998). Histopathologic changes seen in esophagectomy specimens from the high-risk region of Linxian, China: potential clues to an etiologic exposure?. Hum. Pathol..

[63] Kamangar F., Strickland P.T., Pourshams A., Malekzadeh R., Boffetta P., Roth M.J., Abnet C.C., Saadatian-Elahi M., Rakhshani N., Brennan P. (2005). High exposure to polycyclic aromatic hydrocarbons may contribute to high risk of esophageal cancer in northeastern Iran. Anticancer Res..

[64] Preussmann R., Habs M., Habs H., Stununeyer D.  (1983). Fluoro-substituted N-nitrosamines. 6. Cardnogenidty of *N*-nitroso-(2,2,2-trifluoroethyl)-ethylamine in rats. Carcinogenesis.

[65] Lin K., Shen W., Shen Z., Wu Y., Lu S. (2002). Dietary exposure and urinary excretion of total N-nitroso compounds, nitrosamino acids and volatile nitrosamine in inhabitants of high- and low-risk areas for esophageal cancer in southern China. Int. J. Cancer.

[66] Jakszyn P., Gonzalez C.A. (2006). Nitrosamine and related food intake and gastric and esophageal cancer risk: a systematic review of the epidemiological evidence. World J. Gastroenterol..

[67] Munoz N., Crespi M., Grassi A., Qing W.G., Qiong S., Cai L.Z. (1982). Precursor lesions of esophageal cancer in high-risk populations in Iran and China. Lancet.

[68] Crespi M., Munoz N., Grassi A., Aramesh B., Amiri G., Mojtabai A., Casale V. (1979). Esophageal lesions in northern Iran: a premalignant condition?. Lancet.

[69] Jaskiewicz K., Venter F.S., Marasas W.F. (1987). Cytopathology of the esophagus in Transkei. J. Natl. Cancer Inst..

[70] Wang G.Q., Abnet C.C., Shen Q., Lewin K.J., Sun X.D., Roth M.J., Qiao Y.L., Mark S.D., Dong Z.W., Taylor P.R. (2005). Histological precursors of esophageal squamous cell carcinoma: results from a 13 year prospective follow up study in a high risk population. Gut.

[71] Labenz J., Jaspersen D., Kulig M., Leodolter A., Lind T., Meyer-Sabellek W., Stolte M., Vieth M., Willich S., Malfertheiner P. (2004). Risk factors for erosive esophagitis: a multivariate analysis based on the ProGERD study initiative. Am. J. Gastroenterol..

[72] Jacob J.H., Riviere A., Mandard A.M., Munoz N., Crespi M., Etienne Y., Castellsague X., Marnay J., Lebigot G., Qiu S.L. (1993). Prevalence survey of precancerous lesions of the esophagus in a high-risk population for esophageal cancer in France. Eur. J. Cancer Prev..

[73] Brucher B.L., Stein H.J., Bartels H., Feussner H., Siewert J.R. (2001). Achalasia and esophageal cancer: incidence, prevalence, and prognosis. World J. Surg..

[74] Zaninotto G., Rizzetto C., Zambon P., Guzzinati S., Finotti E., Costantini M. (2008). Long-term outcome and risk of esophageal cancer after surgery for achalasia. Br. J. Surg..

[75] Kim Y.T., Sung S.W., Kim J.H. (2001). Is it necessary to resect the diseased esophagus in performing reconstruction for corrosive esophageal stricture?. Eur. J. Cardiothorac. Surg..

[76] Kochhar R., Sethy P.K., Kochhar S., Nagi B., Gupta N.M. (2006). Corrosive induced carcinoma of esophagus: report of three patients and review of literature. J. Gastroenterol. Hepatol..

[77] Appelqvist P., Salmo M. (1980). Lye corrosion carcinoma of the esophagus: a review of 63 cases. Cancer.

[78] Akbari M.R., Malekzadeh R., Nasrollahzadeh D., Amanian D., Sun P., Islami F., Sotoudeh M., Semnani S., Boffeta P., Dawsey S.M. (2006). Familial risks of esophageal cancer among the Turkmen population of the Caspian littoral of Iran. Int. J. Cancer.

[79] Chen X.B., Chen G.L., Liu J.N., Yang J.Z., Yu D.K., Lin D.X., Tan W. (2009). [Genetic polymorphisms in STK15 and MMP-2 associated susceptibility to esophageal cancer in Mongolian population]. Zhonghua Yu Fang Yi Xue Za Zhi.

[80] Druesne-Pecollo N., Tehard B., Mallet Y., Gerber M., Norat T., Hercberg S., Latino-Martel P. (2009). Alcohol and genetic polymorphisms: effect on risk of alcohol-related cancer. Lancet Oncol..

[81] Lao-Sirieix P., Caldas C., Fitzgerald R.C. (2010). Genetic predisposition to gastro-esophageal cancer. Curr. Opin. Genet. Dev..

[82] Lewis S.J., Smith G.D. (2005). Alcohol, ALDH2, and esophageal cancer: a meta-analysis which illustrates the potentials and limitations of a Mendelian randomization approach. Cancer Epidemiol. Biomarkers Prev..

[83] Dong L.M., Potter J.D., White E., Ulrich C.M., Cardon L.R., Peters U. (2008). Genetic susceptibility to cancer: the role of polymorphisms in candidate genes. JAMA.

[84] Li H., Borinskaya S., Yoshimura K., Kal'ina N., Marusin A., Stepanov V.A., Qin Z., Khaliq S., Lee M.Y., Yang Y. (2009). Refined geographic distribution of the oriental ALDH2*504Lys (nee 487Lys) variant. Ann. Hum. Genet..

[85] Cui R., Kamatani Y., Takahashi A., Usami M., Hosono N., Kawaguchi T., Tsunoda T., Kamatani N., Kubo M., Nakamura Y. (2009). Functional variants in ADH1B and ALDH2 coupled with alcohol and smoking synergistically enhance esophageal cancer risk. Gastroenterology.

[86] Ma W.J., Lv G.D., Zheng S.T., Huang C.G., Liu Q., Wang X., Lin R.Y., Sheyhidin I., Lu X.M. (2010). DNA polymorphism and risk of esophageal squamous cell carcinoma in a population of North Xinjiang, China. World J. Gastroenterol..

[87] Zhuo W.L., Zhang Y.S., Wang Y., Zhuo X.L., Zhu B., Cai L., Chen Z.T. (2009). Association studies of CYP1A1 and GSTM1 polymorphisms with esophageal cancer risk: evidence-based meta-analyses. Arch. Med. Res..

[88] Esquela-Kerscher A., Slack F.J. (2006). Oncomirs [mdash] microRNAs with a role in cancer. Nat. Rev. Cancer.

[89] Zhou S.L., Wang L.D. (2010). Circulating microRNAs: novel biomarkers for esophageal cancer. World J. Gastroenterol..

[90] Sugito N., Ishiguro H., Kuwabara Y., Kimura M., Mitsui A., Kurehara H., Ando T., Mori R., Takashima N., Ogawa R. (2006). RNASEN regulates cell proliferation and affects survival in esophageal cancer patients. Clin. Cancer Res..

[91] Gammon M., Schoenberg J., Ahsan H., Risch H., Vaughan T., Chow W., Rotterdam H., West A., Dubrow R., Stanford J. (1997). Tobacco, alcohol, and socioeconomic status and adenocarcinomas of the esophagus and gastric cardia. J. Natl. Cancer Inst..

[92] El-Serag H., Hill C., Jones R. (2009). Systematic review: the epidemiology of gastro-esophageal reflux disease in primary care, using the UK General Practice Research Database. Aliment. Pharmacol. Ther..

[93] Garcia Rodriguez L.A., Lagergren J., Lindblad M. (2006). Gastric acid suppression and risk of esophageal and gastric adenocarcinoma: a nested case control study in the UK. Gut.

[94] Solaymani-Dodaran M., Logan R.F., West J., Card T., Coupland C. (2004). Risk of esophageal cancer in Barrett's esophagus and gastro-esophageal reflux. Gut.

[95] Ruigomez A., Garcia Rodriguez L.A., Wallander M.A., Johansson S., Graffner H., Dent J. (2004). Natural history of gastro-esophageal reflux disease diagnosed in general practice. Aliment. Pharmacol. Ther..

[96] Brown L.M., Devesa S.S. (2002). Epidemiologic trends in esophageal and gastric cancer in the United States. Surg. Oncol. Clin. N. Am..

[97] Haggitt R.C., Tryzelaar J., Ellis F.H., Colcher H. (1978). Adenocarcinoma complicating columnar epithelium-lined (Barrett's) esophagus. Am. J. Clin. Pathol..

[98] Kim R., Weissfeld J.L., Reynolds J.C., Kuller L.H. (1997). Etiology of Barrett's metaplasia and esophageal adenocarcinoma. Cancer Epidemiol. Biomarkers Prev..

[99] Miwa K., Sahara H., Segawa M., Kinami S., Sato T., Miyazaki I., Hattori T. (1996). Reflux of duodenal or gastro-duodenal contents induces esophageal carcinoma in rats. Int. J. Cancer.

[100] Chow W.-H., Blaser M.J., Blot W.J., Gammon M.D., Vaughan T.L., Risch H.A., Perez-Perez G.I., Schoenberg J.B., Stanford J.L., Rotterdam H. (1998). An Inverse Relation between cagA+ Strains of Helicobacter pylori Infection and Risk of Esophageal and Gastric Cardia Adenocarcinoma. Cancer Res..

[101] Ye W., Held M., Lagergren J., Engstrand L., Blot W.J., McLaughlin J.K., Nyren O. (2004). Helicobacter pylori Infection and Gastric Atrophy: Risk of Adenocarcinoma and Squamous-Cell Carcinoma of the Esophagus and Adenocarcinoma of the Gastric Cardia. J. Natl. Cancer Inst..

[102] Wu A.H., Crabtree J.E., Bernstein L., Hawtin P., Cockburn M., Tseng C.-C., Forman D. (2003). Role of <I>Helicobacter pylori</I> CagA+ strains and risk of adenocarcinoma of the stomach and esophagus. Int. J. Cancer.

[103] El-Omar E.M. (2001). The importance of interleukin 1beta in Helicobacter pylori associated disease. Gut.

[104] Shaheen N.J., Crosby M.A., Bozymski E.M., Sandler R.S. (2000). Is there publication bias in the reporting of cancer risk in Barrett's esophagus?. Gastroenterology.

[105] van der Burgh A., Dees J., Hop W.C., van Blankenstein M. (1996). Esophageal cancer is an uncommon cause of death in patients with Barrett's esophagus. Gut.

[106] O'Connor J.B., Falk G.W., Richter J.E. (1999). The incidence of adenocarcinoma and dysplasia in Barrett's esophagus: report on the Cleveland Clinic Barrett's Esophagus Registry. Am. J. Gastroenterol..

[107] Armstrong D. (2008). Should patients with Barrett's esophagus be kept under surveillance? The case for. Best Pract. Res. Clin. Gastroenterol..

[108] Post P.N., Siersema P.D., Van Dekken H. (2007). Rising incidence of clinically evident Barrett's esophagus in The Netherlands: a nation-wide registry of pathology reports. Scand. J. Gastroenterol..

[109] Jeon J., Luebeck E.G., Moolgavkar S.H. (2006). Age effects and temporal trends in adenocarcinoma of the esophagus and gastric cardia (United States). Cancer Causes Cont..

[110] Kuczmarski R.J., Flegal K.M., Campbell S.M., Johnson C.L. (1994). Increasing Prevalence of Overweight Among US Adults: The National Health and Nutrition Examination Surveys, 1960 to 1991. JAMA.

[111] Morris Brown L., Swanson C.A., Gridley G., Swanson G.M., Schoenberg J.B., Greenberg R.S., Silverman D.T., Pottern L.M., Hayes R.B., Schwartz A.G. (1995). Adenocarcinoma of the Esophagus: Role of Obesity and Diet. J. Natl. Cancer Inst..

[112] Vaughan T.L., Davis S., Kristal A., Thomas D.B. (1995). Obesity, alcohol, and tobacco as risk factors for cancers of the esophagus and gastric cardia: adenocarcinoma *versus* squamous cell carcinoma. Cancer Epidemiol. Biomarkers Prev..

[113] Lindblad M., Rodriguez L.A., Lagergren J. (2005). Body mass, tobacco and alcohol and risk of esophageal, gastric cardia, and gastric non-cardia adenocarcinoma among men and women in a nested case-control study. Cancer Causes Cont..

[114] Lagergren J., Ye W., Bergstrom R., Nyren O. (2000). Utility of Endoscopic Screening for Upper Gastrointestinal Adenocarcinoma. JAMA.

[115] Cook M.B., Greenwood D.C., Hardie L.J., Wild C.P., Forman D. (2008). A systematic review and meta-analysis of the risk of increasing adiposity on Barrett's esophagus. Am. J. Gastroenterol..

[116] Rubenstein J.H., Kao J.Y., Madanick R.D., Zhang M., Wang M., Spacek M.B., Donovan J.L., Bright S.D., Shaheen N.J. (2009). Association of adiponectin multimers with Barrett's esophagus. Gut.

[117] Vaughan T.L., Kristal A.R., Blount P.L., Levine D.S., Galipeau P.C., Prevo L.J., Sanchez C.A., Rabinovitch P.S., Reid B.J. (2002). Nonsteroidal anti-inflammatory drug use, body mass index, and anthropometry in relation to genetic and flow cytometric abnormalities in Barrett's esophagus. Cancer Epidemiol. Biomarkers Prev..

[118] Eng C., Spechler S.J., Ruben R., Li F.P. (1993). Familial Barrett esophagus and adenocarcinoma of the gastroesophageal junction. Cancer Epidemiol. Biomarkers Prev..

[119] Chak A., Ochs-Balcom H., Falk G., Grady W.M., Kinnard M., Willis J.E., Elston R., Eng C. (2006). Familiality in Barrett's Esophagus, Adenocarcinoma of the Esophagus, and Adenocarcinoma of the Gastroesophageal Junction. Cancer Epidemiol. Biomarkers Prev..

[120] Feber A., Xi L., Luketich J.D., Pennathur A., Landreneau R.J., Wu M., Swanson S.J., Godfrey T.E., Litle V.R. (2008). MicroRNA expression profiles of esophageal cancer. J. Thorac. Cardiovasc. Surg..

[121] Yang H., Gu J., Wang K.K., Zhang W., Xing J., Chen Z., Ajani J.A., Wu X. (2009). MicroRNA expression signatures in Barrett's esophagus and esophageal adenocarcinoma. Clin. Cancer Res..

